# Coronary artery calcification score in migraine patients

**DOI:** 10.1038/s41598-019-50660-9

**Published:** 2019-10-01

**Authors:** Filipp M. Filippopulos, Florian Schoeberl, Hans-Christoph Becker, Sandra Becker-Bense, Ozan Eren, Andreas Straube, Alexander Becker

**Affiliations:** 1 Department of Neurology, Klinikum Grosshadern, Ludwig Maximilians University, Marchioninistrasse 15, 81377 Munich, Germany; 2 Department of Radiology, Klinikum Grosshadern, Ludwig Maximilians University, Marchioninistrasse 15, 81377 Munich, Germany; 3 Department of Radiology, Stanford Hospital & Clinics, 300 Pasteur Drive S072B, Stanford, California 94305 USA; 4 German Center for Vertigo and Balance Disorders, Klinikum Grosshadern, Ludwig Maximilians University, Marchioninistrasse 15, 81377 Munich, Germany; 5 Department of Cardiology, Klinikum Grosshadern, Ludwig Maximilians University, Marchioninistrasse 15, 81377 Munich, Germany

**Keywords:** Migraine, Calcification

## Abstract

Epidemiological studies have shown an increased risk of cardiovascular events in migraineurs. The pathophysiological mechanisms of this observation remain largely unknown. Recent genetic and epidemiologic studies suggest, that atherosclerosis might be the overlapping pathophysiological mechanism in migraine and coronary heart disease. The aim of the present study was to evaluate if the increased cardiovascular risk in migraineurs is attributed to an increased coronary artery calcification. For this the coronary artery calcium score was assessed by computed tomography of the heart in 1.437 patients of which 337 were migraineurs. All patients had a similar cardiovascular risk profile, so that the risk for coronary calcifications could be considered similar between migraineurs and non-migraineurs. The results showed no significant differences in the amount of coronary calcifications in patients with or without migraine. This suggests that a more pronounced coronary artery calcification, as a surrogate marker of coronary atherosclerosis, does not underlie the increased cardiovascular risk in migraineurs. A distinct common pathophysiological mechanism in migraine and coronary heart disease such as endothelial dysfunction or vasospasm should be discussed instead. However, it has to be considered, that the coronary artery calcification score does not indicate the total risk of atherosclerotic changes in the coronary arteries.

## Introduction

A notable number of previous studies have shown an increased comorbidity of patients with migraine, especially migraine with aura, and several different cardiovascular disorders^1,2^. The associations of migraine with deep white matter lesions and the rarer phenomenon of migrainous cerebral infarcts point towards a prominent role of the vasculature in the pathophysiology of migraine^3,4^. While an association between migraine and cerebral ischemic events has been consistently shown^5,6^, results on an association of migraine and myocardial ischemia are quite controversial^1,2,7–9^.

So far, the pathophysiological mechanisms underlying the increased risk of cardiovascular events in migraineurs are not exactly understood. One possible explanation might be a genetic predisposition for vascular dysfunction, which could result in an increased risk for migraine as well as cardiovascular events. Indeed, migraine shows significant overlap with small vessel diseases and white matter lesions, with Cerebral Autosomal Dominant Arteriopathy with Subcortical Infarcts and Leukoencephalopathy (CADASIL) being the model disease^10,11^. A recent meta-analysis of a large number of genome-wide association (GWA) studies including 375.000 subjects revealed a significant number of gene loci associated with migraine, coding for different proteins of blood vessels and smooth muscle tissue^12^. Among these genes are several ones, that specifically increase the risk for migraine and coronary heart disease (CHD), but also genes important for endothelial function, suggesting common biological processes contributing to both diseases^13^.

Coronary calcifications have shown to be a sensitive marker of advanced coronary atherosclerosis^14,15^. Furthermore, the amount of coronary calcifications correlates with the extent of coronary atherosclerosis and to some extent also coronary stenosis^16,17^ and there is a significant association of coronary calcifications with future cardiovascular events^18,19^. One well established approach to assess coronary calcifications with low radiation exposure is measurement of the coronary artery calcium score (CACS; Agatston score) by non-contrast enhanced computed tomography of the heart (CCT). This technique constitutes a fast and non-invasive tool that has been consistently shown to predict the risk of coronary heart disease with high accuracy^20–22^. However, a relevant limitation of this approach is, that it does not directly assess non-calcified coronary plaques and coronary stenosis, thus possibly underestimating the extent of coronary heart disease. Previous studies have shown, that about one to two percent of patients with no coronary calcification at all have considerable coronary atherosclerosis due to non-calcified plaques^23,24^. On the other hand, the negative predictive value of coronary artery calcifications is very high and lies between 93 and 99%^24^, which indicates an overall good estimate of coronary atherosclerosis despite its aforementioned limitations.

To further investigate the common pathophysiological mechanism in migraine and coronary heart disease, as well as to clarify the association of migraine with an increased risk of cardiac events, we performed a CCT and determined the coronary artery calcium score (CACS) in migraineurs and non-migraineurs with a similar cardiovascular risk profile.

## Methods

### Patients

From July 2002 until December 2011, 1.437 Caucasian patients were included in the study. The patients underwent coronary artery calcium (CAC) scanning due to suspected coronary artery disease (CAD). Each patient signed written informed consent before CAC-scanning. The study was approved by the Data Safety Officer and the Ethics Committee of the Medical Faculty of the Ludwig-Maximilians-University Munich.

All methods were performed in accordance with National Institutes of Health guidelines and regulations.

### Identification of migraine patients

Within this collective, patients suffering from migraine were identified according to the German translation of the second edition of the International classification of headache disorders (ICHD-II)^25^. In particular, all subjects were initially questioned on their general headache history. Subjects reporting on at least one headache episode in the last 12 months, independent of the headache character, duration or accompanying symptoms, were considered having a positive headache history. These patients were then screened with a detailed questionnaire which included the diagnostic criteria of migraine as described in the ICHD-II^25^. The final headache classification into migraine or other primary or secondary headache disorders was conducted by a trained physician. Migraineurs with and without aura were pooled together for the statistical analysis.

### Cardiovascular risk factors

Common risk factors for cardiovascular disease were evaluated for all patients by an interview with a trained physician and by reviewing the medical records of all patients. In addition, following parameters were ascertained from each patient in the fasting state: arterial blood pressure, LDL-/HDL-cholesterol levels, triglyceride level and blood glucose level. Diabetes was defined as a fasting blood glucose level >110 mg/dl or treatment with a glucose-lowering agent; hypercholesterolemia was defined as a total cholesterol level >200 mg/dl or treatment with a lipid-lowering medication; hypertension was determined as a systolic blood pressure >140 mmHg or a diastolic value >90 mmHg; smoking was considered a risk factor when there was a positive smoking history within the previous 10 years.

Finally, the body mass index (BMI), defined as the body mass divided by the square of the body height, was assessed in all patients.

### Coronary artery calcium scanning

For CAC scanning a Siemens multislice CT scanner (Somatom Sensation 16, Siemens Medical Solutions, Forchheim Germany) was used in the high-resolution mode. The images acquired were ECG-triggered with a duration of 100 ms. The images were captured at 80% of the R-R interval and while being in an end-inspiratory breath-holding phase. The slices were of 3 mm thickness and a total of 40 slices were obtained to cover the whole heart. Coronary calcifications were automatically recognized when more than four adjacent pixels showed a density greater than 130 Houndsfield units (HU). To quantify the coronary calcium, the Agatston score was computed^26^. The Agatston score is the product of the lesion´s surface area and a weighting factor between one and four, which is being assigned according to the peak density of the lesion. Segments with coronary stents were excluded from analysis.

### Statistical analysis

For statistical analysis the SPSS software package (version 18.0, SPSS Inc. Chicago, Illinois) was used. Group comparisons for continuous data were calculated with a 2-tailed unpaired Student t-test. Categorical data was compared using a x^2^ or fisher exact test. Values were expressed as mean score ± standard deviation (SD); except where indicated. As the distribution of CAC was skewed and scores of zero occurred, CAC was log transformed. The multivariate Cox proportional hazard model was used to obtain risk ratio estimates and 95% confidence intervals for cardiovascular risk factors, migraine and the presence of CAC. Spearman’s rank correlation coefficient was computed to determine correlations between variables. Effects with p-values smaller than 0.05 were considered significant. All values illustrated in the figures are given as mean ± SD.

### Ethical standards

The study was approved by the Data Safety Officer and the Ethics Committee of the Medical Faculty of the Ludwig-Maximilians-University Munich. Informed consent was obtained from all participants included in the study.

## Results

For all included patients (1.437; 954 male, 483 female) an adequate image quality was obtained for the analysis of CAC. The mean age of the patients was 58.4 ± 21.8 years. There were no significant differences regarding age between men (mean age: 56.3 ± 20.6 years) and women (mean age: 62.5 ± 21.1 years; p = 0.3). 56.8% of all the patients in our cohort suffered from arterial hypertension, 32.0% had hyperlipidemia, 33.0% had type 2 diabetes and 30.3% were smokers. Out of all patients, 23.5% suffered from migraine. The cardiovascular risk factors (triglycerides, hypertension, smoking, HbA1c-value, diabetes) did not differ between patients with and without migraine (Table 1).

**Table 1:** Baseline characteristics of the whole cohort. n: number.

	All	Women	Men
Number n	1437	483	954
Age years	58.4 ± 21.8	62.5 ± 21.1	56.3 ± 20.6
Migraine n (%)	337 (23.5)	152 (31.5)	185 (19.4)
Hypertension n (%)	830 (57.8)	249 (51.6)	581 (60.9)
Hyperlipidemia n (%)	460 (32.0)	118 (24.4)	342 (35.8)
Diabetes n (%)	373 (25.6)	116 (24.4)	257 (26.9)
Smoking n (%)	402 (28.0)	121 (25.1)	281 (29.5)

### Relation of CAC Score, cardiovascular risk factors and migraine

The mean CAC-score (CACS) across the whole study population was 188 ± 133 and showed a positive correlation with the factor age. As expected, younger patients showed a lower CACS, while the CACS increased in older patients. For example, the CACS was 5.1 ± 7.2 in patients younger than 30 years and 571 ± 627 in patients older than 70 years (p < 0.001).

When comparing the CACS in patients with and without cardiovascular risk factors, a significantly higher (p < 0.001) CACS was found in patients with a present cardiovascular risk factor: smokers had a CACS of 237 ± 201, patients with hypertension 289 ± 223, hypercholesterolemia 620 ± 455 and diabetes 712 ± 566, while patients without any risk factors had a CACS of 95 ± 51. The CACS did not differ between migraineurs (men: 188 ± 97, women: 109 ± 66) and those patients without migraine (men: 172 ± 88, women: 117 ± 70) (p = 0.21) (Table 2).

**Table 2:** The cardiovascular risk profile of patients with and without migraine in detail. n: number, BMI: body mass index, LDL-C.

	Men		p-value	women		p- value
	migraine	no migraine		migraine	no migraine	
Number n	185	769		152	331	
BMI kg/m²	25.8 ± 5.9	26.3 ± 6.1	*0.23*	26.2 ± 6.1	26.4 ± 6.2	*0.28*
Age (years)	55.4 ± 18.8	56.5 ± 19.9	*0.4*	60.8 ± 20.0	63.2 ± 20.1	*0.17*
LDL-C (mg/dl)	119 ± 37	118 ± 40	*0.37*	107 ± 37	105 ± 30	*0.22*
HDL-C (mg/dl)	47 ± 27	49 ± 30	*0.44*	45 ± 24	46 ± 27	*0.31*
Statin use n (%)	69 (37.2)	273 (35.5)	*0.29*	37 (24.3)	81 (24,5)	*0.19*
Triglyceride (mg/dl)	160 ± 99	158 ± 94	*0.29*	145 ± 86	143 ± 87	*0.21*
Hypertension n (%)	105 (56.8)	476 (61.9)	*0.2*	78 (51.3)	171 (51.7)	*0.31*
Smoking n (%)	56 (30.3)	225 (29.3)	*0.21*	40 (26.3)	81 (24.5)	*0.19*
HbA1c (%)	6.3 ± 2.3	6.4 ± 2.3	*0.41*	6.6 ± 2.5	6.4 ± 2.5	*0.23*
Diabetes n (%)	61 (33.0)	196 (25.5)	*0.37*	43 (28.3)	73 (22.1)	*0.27*
CAC Score	188 ± 97	172 ± 88	*0.41*	109 ± 66	117 ± 70	*0.38*

### Multivariate analysis

To further evaluate the correlation between all obtained cardiovascular risk factors, migraine and CACS a multivariate analysis was performed. Age, diabetes, arterial hypertension, and statin therapy could be identified as independent risk factors for the development of CAC in both men and women, while the BMI, smoking, and LDL-C were not associated with CAC. The presence of migraine was also not associated with an increased calcium score (Table 3).

**Table 3:** Multivariate analysis of different risk factors for the presence of CAC.

	Women	p-value	Men	p-value
Age	0.153 ± 0.024	<0.0001*	0.121 ± 0.027	<0.0001*
BMI	0.049 ± 0.028	*0.19*	0.036 ± 0.027	*0.12*
Diabetes	0.123 ± 0.081	<0.001*	0.140 ± 0.10	<0.001*
LDL-C	0.028 ± 0.046	*0.24*	0.027 ± 0.026	*0.23*
Statin use	0.822 ± 0.125	<0.001*	0.890 ± 0.190	<0.001*
Hypertension	0.79 ± 0.103	0.002*	0.82 ± 0.127	0.003*
Smoking	0.038 ± 0.017	*0.60*	0.075 ± 0.031	*0.72*
Migraine	0.035 ± 0.030	*0.16*	0.029 ± 0.011	*0.19*

* indicates a significant difference.

## Discussion

The purpose of our study was to evaluate the impact of migraine on the extent of CAC in a large cohort with similar cardiovascular risk factors. The study also aimed to evaluate if coronary calcification is the pathophysiological substrate of the increased cardiovascular risk in migraineurs. CAC could already be identified as a specific marker of individual atherosclerotic burden in previous studies^16,17^. Even more important, CAC is suitable as a prognostic marker for future cardiovascular events with a high diagnostic accuracy^20–22^. The amount of coronary calcifications has been shown to correlate quite well with the extent of coronary atherosclerosis, i.e. calcified and non-calcified plaques as well as coronary stenosis^14–17^. Even though non-calcified plaques and coronary stenosis cannot directly be assessed by this approach, only one to two percent of patients with zero CAC have been shown to suffer from a significant atherosclerosis^23,24^. Additionally, the negative predictive value of CAC has been shown to lie between 93 and 99%^24^, so that overall CAC was considered to be a validated and reliable tool for evaluating a possible aetiopathological link between migraine and CAC.

In the present study, the population was characteristic for a cohort of a western industrialized country and showed a typical distribution of cardiovascular risk factors. Within the study population a substantial subgroup of patients, in particular 23.5%, suffered from migraine, which correlates well with previous studies on migraine prevalence in Europe^27^. As already described in several studies before^18,28^, there was an increase in CAC with the presence of conventional cardiovascular risk factors. In the performed multivariate analysis an impact of age, diabetes, hypertension, and statin therapy on the development of CAC was found. However, although statin therapy was significantly associated with an increased CAC, the LDL-cholesterol value was not an independent risk factor. A plausible explanation for this might be, that most patients with hypercholesterolemia already received an efficient therapy with statins leading to a reduction of their cholesterol levels. Therefore, statin therapy could be identified as an independent predictor of former elevated CAC. These findings could be observed the same way in both, patients with and without migraine.

However, in the present study migraine had no significant impact on the development of CAC. This was persistent across all age groups and valid for men and women likewise. Thus, an equal atherosclerotic burden could be assumed for both groups (migraineurs and non-migraineurs).

Since some past studies have reported a higher risk of CAD in migraineurs^7,8^, and with CACS predicting CAD^20–22^, it was assumed, that migraine should also be associated with CACS. Furthermore, multigene analyses have revealed an association between migraine and vascular phenotypes^12^, especially via the gene PHACTR1 which seems to be associated with CAD^29^ and CAC^30^. However, according to our findings, migraine does not seem to directly impact CACS in the majority of migraineurs. In turn, this indicates, that additional and separate pathophysiological mechanisms apart from CAC might underlie the increased number of cardiovascular events in patients with migraine, which is in line with some previous findings showing even a lower rate of coronary artery calcification disease in migraineurs^31^. One such common pathophysiological trait could be endothelial dysfunction and/or dysregulation which has been suggested to play a role in the pathophysiology of migraine^32,33^ and CAD likewise^34^. Another different mechanism could be an increased susceptibility to vasospasm in migraine patients as it seems to be the case in patients with the reversible cerebral vasoconstriction syndrome^35^. Especially, in women such segmental vasospasms of the coronary arteries may also not be neglected as an important factor for myocardial ischemia^36^.

Furthermore, it could be even argued that CACS might underestimate the cardiovascular risk profile in patients with migraine and thus is not appropriate to estimate the total risk of CAD in migraine patients sufficiently. To evaluate though the predictive value of CACS in migraineurs further studies are needed. Particularly, we still do not know whether non-calcified plaques of the coronary arteries and/or coronary artery stenosis is more prevalent and more severe in migraineurs. In the present cohort the number of patients who underwent additional coronary angiography was too small, so that we could not address this question by a subgroup analysis.

### Limitations

All patients participating in the present study underwent coronary artery calcification scanning because of a suspected coronary artery disease. Therefore, the study population cannot be considered an unselected population of patients with migraine. Due to this selection bias the prevalence of cardiovascular risk factors and CAC was disproportionately high in our cohort as compared to a selected cohort of migraine patients. One additional limitation is that the applied approach of CAC cannot evaluate the amount of non-calcified coronary plaques and thus might underestimate the extent of coronary atherosclerosis in the examined cohort.

## Conclusion

Migraine does not correlate with an increased coronary artery calcification score in the present cohort; thus, it seems that the increased cardiovascular risk in patients with migraine is not due to a more pronounced atherosclerosis of the coronary arteries. However, a higher amount of non-calcified plaques leading to more prevalent and severe coronary atherosclerosis in migraineurs cannot be sufficiently ruled out, since we did not perform CT angiography of the coronary arteries systematically.

## Data Availability

The data for this study includes personal information, so that it cannot be shared at its current format due to ethical and legal restrictions. Readers are welcome to contact the corresponding author to receive anonymized data upon reasonable request.
